# Adolescent reports of subjective socioeconomic status: An adequate alternative to parent-reported objective and subjective socioeconomic status?

**DOI:** 10.1371/journal.pone.0317777

**Published:** 2025-01-17

**Authors:** Erin K. Davisson, Fernanda C. Andrade, Jennifer Godwin, Rick Hoyle

**Affiliations:** 1 Department of Psychology and Neuroscience, Duke University, Durham, North Carolina, United States of America; 2 Center for Child and Family Policy, Duke University, Durham, North Carolina, United States of America; SUNY Downstate Health Sciences University, UNITED STATES OF AMERICA

## Abstract

Socioeconomic status (SES) is associated with well-being outcomes across studies; however, there is wide variation in its measurement, particularly in adolescence. One key difference in measures of SES concerns whether participants relay objective information—for example, years of education, household income—or subjective perceptions of socioeconomic status, either with or without reference to others or society. Although parents are often considered the best source of SES information—especially objective SES—within families, interviewing parents within the context of adolescent research is costly, time-consuming, and not always feasible. Given the importance of SES for outcomes in adolescence and cumulative effects over the lifespan, we used data from adolescents (*N* = 702) and parents (*N*s = 664–730) to examine whether adolescent reports of SES serve as reasonable proxies for parent reports of both objective and subjective SES, as well as administrative data assessing family SES and neighborhood SES. Consistent with our hypotheses, adolescents’ reports of subjective SES were moderately correlated with parent reports and administrative data tapping family SES. Moreover, adolescents’ reports of subjective SES predicted adolescent-reported measures of well-being, including mental health, physical health, school performance, problem behavior, and alcohol use to the same degree as or better than parent reports of both subjective and objective SES and administrative data. These findings suggest that adolescent reports of subjective SES—using two different, easily understood measures—can stand in as reasonable alternatives to parent-reported SES and administrative data.

## Introduction

Socioeconomic status (SES) is a key determinant of well-being in the United States and around the world, across outcomes as varied as mental health [1, 2], physical health [3, 4], and externalizing problems including substance use [5, 6]. The relationship between socioeconomic status and outcomes is complex, but appears to be especially consequential for adolescents, for whom low SES is a meaningful risk factor for later negative outcomes including lower academic performance [7], poorer physical and mental health [8, 9], and more risky behavior [10]. Consequently, it is imperative that researchers evaluating aspects of well-being account for the impact of SES in their work, especially in adolescence, as the impact of low SES on well-being outcomes accumulates across the lifespan [11].

Although psychological research, including adolescent research, broadly acknowledges the importance of SES for outcomes, there is wide variability in its conceptualization and measurement. Both within and across disciplines and studies, commonly used SES measures vary on a key dimension: the extent to which they are *objective* indicators of economic or material resources—family income or assets, education (or parents’ education), occupational prestige (e.g., [12]), or some combination of the three—or *subjective* perceptions of socioeconomic status, particularly as it relates to others in society or within a particular frame of reference such as one’s community or school [13]. Subjective measures of SES may tap objective indicators to the extent that they capture subjective experiences resulting directly from material resources (e.g., financial security reflects income or other assets to some degree). However, subjective SES also reflects objective SES because social class itself is influenced by one’s material resources in addition to perceptions of one’s relative rank in society as signaled by cultural symbols of worth such as clothing, social behaviors such as language, and comparisons between one’s own rank relative to others over time [14].

Given that researchers use both objective and subjective SES measures in service of the same goal—to characterize participants’ social and economic resources—understanding the relation between the two types of measures is crucial, as measures and proxies may vary in their relevance to target outcomes [15]. Research across disciplines and outcomes finds that objective and subjective SES are moderately-to-strongly, but not perfectly, correlated (see [13], for a meta-analysis). Accordingly, objective and subjective SES have each been shown to predict well-being outcomes, with several studies and meta-analyses demonstrating stronger effects for subjective SES in predicting outcomes including physical health in adulthood [16, 17], adolescent physical and mental health [18], subjective well-being [13], and substance use [19]. As in research linking SES with well-being outcomes in adult samples, adolescent research demonstrates differences in the predictive ability of objective (e.g., administrative data, parent-reported objective, or adolescent self-reported objective) vs. subjective SES measures (e.g., [9]), where subjective measures tend to predict variance in well-being and health outcomes better than objective measures.

The distinction between and relative utility of objective vs. subjective measures of SES becomes emphasized when conducting research with adolescents. Within a family, parents have the best information regarding the true values of the objective SES indicators (e.g., household income), whereas adolescents are unlikely to have direct knowledge of many of these indicators. In adolescent research, certain measures of adolescent-reported objective SES are used commonly and demonstrate moderate yet significant associations with parent reports (e.g., the Family Affluence Scale; [20, 21]), yet many studies do not ask adolescents to report on objective SES, relying instead on adolescent-reported subjective SES alone or in combination with parent reports or administrative data. For researchers who study adolescents, limited funding availability may make interviewing parents as well as adolescents impractical or even impossible; likewise, administrative data are not always available to researchers. Consequently, understanding which subjective, adolescent-reported measures of SES perform best in lieu of parent-reported objective and subjective SES would be valuable for optimizing data collection strategies for adolescent research. Prior work indicates that reports by adolescents—especially older adolescents [22]—perform reasonably well as proxies of parent reports of objective SES indicators (e.g., parental education, parental occupation) [23, 24]. More recent work finds that adolescent-reported subjective SES corresponds reasonably well to parent reports of objective SES indicators [25]. Given the relative importance of subjective SES for predicting adolescent well-being outcomes, we extend this work by examining how well three measures of adolescent-reported subjective SES correspond with parent reports of subjective SES on parallel measures, parent reports of objective SES indicators, and administrative data.

### Overview and hypotheses

We aim to examine the degree to which adolescent reports of subjective SES approximate parent reports of objective SES, parent reports of subjective SES, and administrative data. Specifically, we examine whether adolescents’ reports of socioeconomic status may serve as reasonable proxies for parent reports of SES. We add to the existing literature by leveraging a unique dataset containing multiple indicators of parent reports of objective SES, parent reports of subjective SES, adolescent reports of subjective SES, administrative data on SES indicators, and well-being. We assess the correlations between adolescent reports of subjective SES, parent-reported indicators of subjective and objective SES, and SES reflected in administrative data. In addition, given the importance of subjective SES in predicting well-being outcomes in adolescence, we examine the predictive validity of adolescent reports of subjective SES for well-being outcomes, relative to the other sources of information about SES.

Specifically, we hypothesize that adolescent-reported measures of subjective SES are moderately correlated (*r*s > .25) with parent-reported measures of objective and subjective SES and administrative data. We additionally hypothesize that these adolescent-reported measures of subjective SES predict adolescent-reported measures of well-being, including school performance, physical and mental health, problem behavior, and alcohol use, at least as well as parent-reported measures of objective and subjective SES and administrative data. Finally, we examine whether adolescents’ age affects the degree to which adolescent-reported measures of subjective SES correlate with parent measures and predict well-being outcomes, hypothesizing that older adolescents’ reports of SES are more strongly correlated with parent reports and more predictive of outcomes.

## Method

### Participants

A representative sample of middle school students attending public school in North Carolina were recruited during the 2014–2015 school year for a multi-wave study (*n* = 2,104; see [26] for more details). These analyses use youth-reported data from follow-up Waves 3 (*n* = 702, *M*_age_ = 15.1, *SD* = 1.14, collected from February 7, 2018, through November 27, 2018) and 4 (*n* = 891, *M*_age_ = 17.2, *SD* = 1.18, collected from March 11, 2020-January 4, 2021). In addition, we use parent data collected from parent reporters who were contacted for participation in Waves 2, 3, and 4 (*n*_Wave2_ = 696, collected March 15, 2017-December 18, 2017; *n*_Wave3_ = 664, collected March 2, 2018-December 20, 2018; *n*_Wave4_ = 730, collected April 3, 2020-February 23, 2021). These analyses were limited to adolescents who reported at least one SES variable at Wave 3 (*n* = 702). Table 1 provides descriptive statistics for the adolescent sample with data available at Wave 3, from which predictor variables were collected.

**Table 1:** Descriptive statistics.

	*n*	Mean (*SD*) or Proportion (*n*)	Min	Max
SES—Wave 3				
Subjective SES				
Adolescent-reported	695	1.91 (.69)	0	3
Parent-reported	610	1.70 (.84)	0	3
Subjective Social Status (SSS)				
Adolescent-reported	701	6.58 (1.49)	1	10
Parent-reported	611	6.16 (1.80)	1	10
Food Security				
Adolescent-reported^1^	701	.00 (1.00)	-7.25	.50
Parent-reported^1^	609	.04 (.94)	-4.81	.41
Parental Years of Education (11 = < 12)	666	15.20 (2.69)	11	26
Household Income	644			
Less than $15,000		.04 (26)		
$15,000-$24,999		.10 (64)		
$25,000-$34,999		.10 (62)		
$35,000-$44,999		.13 (85)		
$45,000-$59,999		.12 (78)		
$60,000-$79,999		.20 (126)		
$80,000-$99,999		.11 (71)		
$100,000-$149,999		.11 (71)		
$150,000 or more		.09 (61)		
Economically Disadvantaged	686	.51 (350)		
Neighborhood SES	700	$56,870 ($32,046)	$5,977	$250,000
Adolescent Well-being—Wave 4				
Academic Performance (Grades)	532	8.04 (1.52)	1	10
General Health	553	1.86 (.82)	.33	4
Psychological Distress	554	2.33 (.84)	1	5
Problem Behavior	552	.19 (.31)	0	3.08
Past-Year Alcohol Use	554	.37 (205)		
Adolescent Demographics				
Age at Wave 3	702			
Age ≤ 14		.34 (240)		
Age 15		.29 (205)		
Age ≥ 16		.37 (257)		
Minority	702	.43 (299)		
Female	702	.55 (389)		
Parent Demographics—Wave 3				
Age	610	44.60 (6.91)	28	74
Minority	612	.37 (251)		
Female	614	.90 (595)		

*Note*.

^1^Items for these scales were standardized (*M =* 0, *SD* = 1) prior to creating the mean score, yielding low minimum values due to the relative infrequency of food insecurity in the sample. See S2 Table for additional details.

Descriptive statistics for the Wave 3 sample relative to the baseline sample at Wave 1, and the population from which the sample was drawn appear in S1 Table. Relative to those who did not participate in the Wave 3 follow-up, adolescents who participated were more likely to be female (χ^2^ (1) = 4.66, *p* < .05) and non-Hispanic white (χ^2^ (6) = 27.13, *p* < .01). Participants with available data at the Wave 3 follow-up—and, therefore, included in the present analysis—were more likely to be economically disadvantaged (51.02%) compared to those without available Wave 3 data (37.57%; χ^2^ (1) = 33.68, *p* < .01).

### Procedure

Parents provided their consent for adolescents to participate and their own consent to participate in the parent study; adolescents provided assent. All consent and assent were provided on IRB-approved informed consent (assent) forms via Qualtrics, where participants typed their name and selected “Next” to indicate they would like to participate. Parents and adolescents completed all waves of data collection via Qualtrics. Participants received $30 for each wave of data collection they completed. The Duke University Campus Institutional Review Board approved all study procedures (protocols 2017-0915/B0828 and 2017-0332/D0396).

### Measures

#### Subjective measures of SES

At Wave 3, *adolescent-reported family subjective SES* is based on a single item created for this study: “In your opinion, how is your family doing financially?” rated on a 4-point scale: 3 = *we have enough money to do anything we want*, 2 = *we have all the money we need to live a comfortable life*, 1 = *money is tight*, *but we are able to meet our basic needs*, and 0 = *we do not have enough money to meet basic needs*. In the same wave, *parent-reported subjective SES* was measured by a similar item, “Thinking about the end of each month over the past 12 months, would you say your household generally ended up with”: 3 = *more than enough money left over*, 2 = *some money left over*, 1 = *just enough to make ends meet*, and 0 = *not enough to make ends meet*.

*Adolescent- and parent-reported subjective social status* (SSS) were measured at Wave 3 using the MacArthur Scale of Subjective Social Status [27], a single item assessing perceived social status based on a vertical ladder, where on top (coded 10) are the “people who have the best jobs, lots of money, live in nice places, and go to the best schools,” and at the bottom (coded 1) are the “people who don’t have enough money, don’t live in a nice place, and might not have a job.”

*Adolescent-reported food security* at Wave 3 was measured using a mean score across four standardized items from the National Comorbidity Survey Adolescent Supplement capturing hunger and difficulty obtaining nutritious food over the past 12 months (α = .73; [28]). *Parent-reported food security* was measured at Wave 3 using a mean score across five standardized items from the Food Insecurity Short Form [29] capturing hunger, skipping meals, eating less, and problems obtaining balanced meals due to financial constraints over the past 12 months (α = .83).

#### Objective measures of SES

In Waves 2 through 4, *parent-reported years of education* was assessed by having the parent reporter record the number of years of school he/she completed. At each wave, years completed was recoded to 11 (i.e., equivalent to no high school diploma or GED) if fewer than 12 years were completed. Data were separated into father and mother-reported education based on sex and reported relationship with youth. The majority of variance in years of education across the three waves of available data was between participants (ICC = .88). Therefore, we maximize sample size by first averaging parent reports across waves, and then calculating the maximum across both mother- (*n* = 896) and father-reported (*n* = 122) years of education.

In Waves 2 through 4, *parent-reported household income* was measured by asking parents to report “How much money did your family make in the last month?” This amount was averaged across waves, multiplied by 12 to approximate annual income, and transformed into an 9-category scale: less than $15,000, $15,000-$24,999, $25,000-$34,999, $35,000-$44,999, $45,000-$59,999, $60,000-$79,999, $80,000-$99,999, $100,000-$149,999, and $150,000 or more. As with education, most of the variance in parent-reported household income across waves was between participants (ICC = .81), so we again averaged across waves to maximize available data.

At Wave 1, parents provided consent for survey data to be linked to school administrative data (*n* = 2,048), including *family economic disadvantage*, which was based on eligibility to receive free and/or reduced lunch (coded 0 = *never eligible*, 1 = *ever eligible*) for all years adolescents had available data through the 2014–15 school year (i.e., the school year during which Wave 1 was completed). For analysis, though this variable was collected at Wave 1, we restricted data to adolescents with available data at Wave 3.

We operationalized adolescents’ *neighborhood SES* using median neighborhood income geocoded from American Community Survey 5-year estimates for 2010–2014 [30], based on their home addresses provided at Wave 1 (*n* = 2,099). Though collected at Wave 1, this variable was restricted in analyses to adolescents who had available data at Wave 3.

#### Outcome measures of adolescent well-being

*Adolescent-reported academic performance* was measured at Wave 4 by asking adolescents to report “How have you done in school during the past year?” using a 10-point scale ranging from 1, *All As*, to 10, *Mostly Fs*. Scores were recoded so that higher scores indicated higher levels of academic performance. In previous work [31], this single item correlated well with achievement levels on state end-of-grade tests in math and reading, as well as parent reports of adolescent grades on the same 10-point scale.

We assessed *adolescent-reported general health* at Wave 4 by having adolescents respond to three items adapted from the National Longitudinal Study of Adolescent to Adult Health [32] measuring their general health status and frequency of two types of exercise in the past week. Adolescents answered the question, “In general, how is your health?” on a 0 (*excellent*) to 4 (*poor*) scale. They also reported their frequency of (1) playing an active sport such as basketball, soccer, football, hockey, or swimming and (2) exercise such as walking, jogging, bicycling, running, karate, gymnastics, or dancing on a 0 (*not at all*) to 4 (*every day or more*) scale. These items were recoded and averaged to create a measure of general health for which higher scores indicate better health (α = .52).

*Adolescent-reported psychological distress* was measured at Wave 4 by asking adolescents to complete the 6-item Kessler Psychological Distress scale [33] capturing the frequency he/she had distressing feelings experienced over the past month (i.e., hopelessness, restlessness, depression, worthless). The items were measured on a 5point scale ranging from 1, *none of the time*, to 5, *all of the time*. A mean score was calculated such that high scores indicate greater distress (α = .86).

At Wave 4, we assessed *adolescent-reported problem behavior* by having adolescents report how often they engaged in 26 types of problem behavior over the past month including physical aggression (e.g., pushing another kid), relational aggression (e.g., starting a fight between others) and deviant behavior (e.g., stealing or damaging property), using items from the Problem Behavior Frequency Scale [34]. Frequency was captured on a 6-point scale ranging from 0 = *never*, to 5 = *20 or more times*. An average score across items was calculated with higher scores indicating more frequent problem behavior (α = .93).

We assessed *adolescent-reported alcohol use* at Wave 4, where adolescents respond to a single item about their past-year alcohol use, “On how many occasions in the last year have you had alcoholic beverages to drink, more than just a few sips?” on a 7-point scale ranging from *0 occasions/none* to *40 or more occasions* (adapted from the Monitoring the Future Study; [35]). Responses were recoded into 0 = *no past-year alcohol use* and 1 = *any past-year alcohol use*.

## Analytic plan

To assess the extent to which adolescent-reported measures of subjective SES at Wave 3 (subjective SES, subjective social status, and food security) are suitable proxies for parent-reported objective (parental years of education and household income) and subjective (subjective SES, subjective social status, and food security) measures of SES, we examined bivariate correlations. We also compared correlations between three age groups using age at Wave 3 (age 14 and younger, age 15, and age 16 and older). Using recommendations provided by Gignac and Szodorai [36], which update Cohen’s [37] conventions for interpreting the magnitude of correlation coefficients in behavioral sciences, we considered *r*s in the ranges of .10-.15, .20-.25, and .30-.35 to be small, medium, and large correlations, respectively.

To compare adolescent-reported measures of subjective SES with parent-reported objective and subjective measures of SES as predictors of adolescent-reported well-being, we compare *R*^*2*^ values from regressions predicting the Wave 4 measures of well-being. All models control for sex (coded 0 = *male*, 1 = *female*) and race/ethnicity (coded 0 = *non-Hispanic white*, else 1). For each of the three adolescent-reported SES measures and Wave 4 outcomes we compare the *R*^*2*^ values across models with five different specifications for SES: adolescent-reported subjective SES, matching parent-reported subjective SES, parent-reported income, and parent-reported years of education. To determine whether age of the adolescent reporter affects the proportion of variance of each adolescent well-being outcome accounted for by the different measures of SES, we estimate multi-group regressions for the three age groups described above. For all outcomes except alcohol use, we ran linear regressions in *Mplus* using the MLR estimator and full-information maximum likelihood (FIML) to account for missing data. For alcohol use, we used a logit link regression model with FIML. All analyses limit the sample to adolescents who completed the Wave 3 interview. Data, except for the economic disadvantage variable, are available at https://osf.io/x8psk/?view_only=b4f2730d590a4545be82276bfb21409d.

## Results

### Are adolescent reports of subjective SES reasonable proxies for parent reports?

We first examined bivariate correlations between adolescent-reported subjective SES measures, parent-reported subjective SES measures, parent-reported objective SES measures, and administrative data assessing family economic disadvantage and neighborhood SES to test whether adolescent-reported subjective SES could serve as a proxy for parent-reported subjective measures and objective measures of SES. As seen in Table 2, we observed strong correlations between adolescent-reported *subjective SES* and both parent-reported subjective SES (*r* = .43) and parent-reported household income (*r* = .44). Adolescent-reported subjective SES was moderately-to-strongly correlated with parent-reported subjective social status, food security, parental years of education, family economic disadvantage, and neighborhood SES (*r*s ranged from .25 to .37).

**Table 2:** Pearson correlation coefficients (n) between SES variables.

	Adolescent-Reported			Parent-Reported					Administrative	
	1	2	3	4	5	6	7	8	9	10
1. Subjective SES: Adolescent	1									
	(695)									
2. Subjective Social Status: Adolescent	.51**	1								
	(694)	(701)								
3. Food Security: Adolescent	.32**	.26**	1							
	(695)	(701)	(702)							
4. Subjective SES: Parent	.43**	.35**	.19**	1						
	(605)	(610)	(610)	(610)						
5. Subjective Social Status: Parent	.37**	.45**	.21**	.51**	1					
	(606)	(611)	(611)	(607)	(611)					
6. Food Security: Parent	.29**	.22**	.35**	.36**	.30**	1				
	(604)	(609)	(609)	(607)	(605)	(609)				
7. Parental Years of Education	.29**	.30**	.21**	.31**	.38**	.23**	1			
	(659)	(665)	(666)	(607)	(608)	(606)	(666)			
8. Household Income	.44**	.42**	.27**	.51**	.46**	.38**	.51**	1		
	(637)	(644)	(644)	(591)	(591)	(590)	(642)	(644)		
9. Family Economic Disadvantage	.33**	.29**	.28**	.36**	.33**	.31**	.50**	.58**	1	
	(679)	(685)	(686)	(595)	(595)	(593)	(651)	(629)	(686)	
10. Neighborhood SES	.25**	.29**	.15**	.32**	.31**	.20**	.34**	.48**	.44**	1
	(693)	(699)	(700)	(608)	(609)	(607)	(664)	(642)	(684)	(700)

*Note*.

** *p* < .01.

Similarly, adolescent-reported *subjective social status* was strongly correlated with parent-reported subjective social status (*r* = .45) and parent-reported household income (*r* = .42), and moderately-to-strongly correlated with parent-reported subjective SES, food security, parental years of education, family economic disadvantage, and neighborhood SES (*r*s ranged from .22 to .35). Finally, we observed small-to-medium correlations between adolescent-reported *food security* and all parent-reported subjective SES measures, parent-reported objective SES measures, and variables extracted from administrative data (*r*s ranged from .15 to .28), apart from parent-reported food security, which had a larger correlation (*r* = .35).

### Are older adolescents better proxies for parent reports?

As seen in Table 3, for adolescent-reported *subjective SES*, the patterns of correlation from the full sample were replicated for adolescents age 14 and younger and for those age 16 and older, but not for those age 15. We observed similar strong correlations between adolescent-reported subjective SES and parent-reported subjective SES for the younger and older adolescents (*r*s = .48 and .49, respectively), and a more moderate correlation for the middle group (*r* = .31). A similar pattern emerged for parent-reported subjective social status (*r*s = .40 and .42 for the youngest and oldest adolescents; *r* = .26 for the middle group) and parent-reported household income (*r*s = .50 and .47 for the youngest and oldest adolescents; *r* = .34 for the middle group). Finally, we observed a similar pattern—whereby the youngest and oldest adolescents showed medium-to-large correlations, with more moderate correlations among the middle group—for parent-reported food security (*r*s = .32 and .30 for the youngest and oldest adolescents; *r* = .21 for the middle group), years of parental education (*r*s = .35 and .30 for the youngest and oldest adolescents; *r* = .21 for the middle group), family economic disadvantage (*r*s = .38 and .32 for the youngest and oldest adolescents; *r* = .31 for the middle group), and neighborhood SES (*r*s = .32 and .25 for the youngest and oldest adolescents; *r* = .15 for the middle group).

**Table 3:** Pearson correlation coefficients (n) between SES variables by age group.

	Adolescent-Reported		Parent-Reported					Administrative	
	SSS	Food Security	Subj. SES	SSS	Food Security	Parent Edu. Yrs	HHI	Family Ec. Disadv.	Neigh. SES
Age ≤ 14									
Subjective SES	.50**	.29**	.48**	.40**	.32**	.35**	.50**	.38**	.32**
	(236)	(237)	(208)	(210)	(208)	(229)	(224)	(235)	(237)
SSS		.21**	.37**	.48**	.20**	.30**	.43**	.29**	.33**
		(239)	(210)	(212)	(210)	(231)	(227)	(237)	(239)
Food Security			.21**	.17*	.29**	.23**	.38**	.28**	.19**
			(210)	(212)	(210)	(232)	(227)	(238)	(240)
Age 15									
Subjective SES	.51**	.33**	.31**	.26**	.21**	.21**	.34**	.31**	.15**
	(202)	(202)	(180)	(180)	(180)	(194)	(186)	(195)	(202)
SSS		.31**	.28**	.40**	.15*	.30**	.40**	.30**	.28**
		(205)	(182)	(182)	(182)	(197)	(189)	(198)	(205)
Food Security			.16*	.27**	.19**	.25**	.13	.29**	.14**
			(182)	(182)	(182)	(197)	(189)	(198)	(205)
Age ≥ 16									
Subjective SES	.53**	.33**	.49**	.42**	.30**	.30**	.47**	.32**	.25**
	(256)	(256)	(217)	(216)	(216)	(236)	(227)	(249)	(254)
SSS		.24**	.41**	.45**	.28**	.29**	.44**	.31**	.25**
		(257)	(218)	(217)	(217)	(237)	(228)	(250)	(255)
Food Security			.21**	.19**	.50**	.16*	.30**	.26**	.13**
			(218)	(217)	(217)	(237)	(228)	(250)	(255)

*Note*.

** *p* < .01,

* *p* < .05.

Subj. SES = subjective SES; SSS = subjective social status; Parent Edu. Yrs = parental years of education; HHI = household income; Family Ec. Disadv. = family economic disadvantage; Neigh. SES = neighborhood SES.

For adolescent-reported *subjective social status*, the patterns of correlation from the full sample were generally replicated for all age groups. Specifically, adolescent-reported *subjective social status* was strongly correlated with parent-reported subjective social status (*r*s ranged from .40 to .48), parent-reported subjective SES (*r*s = .28-.41), and parent-reported household income (*r*s = .40-.44). Across age groups, we observed moderate correlations between adolescent-reported *subjective social status* and years of education, family economic disadvantage, and neighborhood SES (*r*s ranged from .25-.33). Finally, we observed small-to-medium correlations between adolescent-reported *subjective social status* and parent-reported food security (*r*s = .15-.28).

As in the full sample, across the age groups, we observed mostly small-to-medium correlations between adolescent-reported *food security* and all parent-reported subjective and objective measures of SES, as well as administrative data (*r*s ranged from .13 to .30), with a few exceptions. Among those age 16 and older, adolescent-reported *food security* was strongly correlated with parent-reported food security (*r* = .50) and was strongly correlated with parent-reported household income (*r* = .38) for adolescents aged 14 and younger.

### How well do adolescent reports of subjective SES predict outcomes?

Next, we examined the extent to which adolescent reports of subjective SES predicted variance in adolescent well-being outcomes—grades, general health, psychological distress, problem behavior, and alcohol use—compared to parent-reported subjective and objective SES measures and administrative data. Bivariate correlations between all SES variables and well-being outcomes appear in S4 Table. All regression models included covariates for sex (coded 0 = *male*, 1 = *female*) and race/ethnicity (coded 0 = *non-Hispanic white*, else 1).

Table 4 displays *R*^*2*^ values for regression models predicting Wave 4 adolescent outcomes from each of the adolescent-reported SES measures, the parent-reported subjective and objective SES measures, administrative data quantifying family and neighborhood SES, as well as a model including only the sex and race/ethnicity covariates.

**Table 4:** *R*^*2*^ (SE) for models predicting Wave 4 outcomes using different measures of SES.

SES Measure	Grades	General Health	Psychological Distress	Problem Behavior	Past-Year Alcohol Use
Adolescent-Reported (Wave 3)					
Subjective SES: Adolescent	.09** (.02)	.08** (.02)	.06** (.02)	.01 (.01)	.03 (.02)
Subjective Social Status: Adolescent	.07** (.02)	.09** (.02)	.06** (.02)	.01 (.01)	.02 (.01)
Food Security: Adolescent	.06** (.02)	.06** (.02)	.06** (.02)	.06 (.05)	.03 (.02)
Parent-Reported (Wave 3)					
Subjective SES: Parent	.08** (.02)	.07** (.02)	.04** (.02)	.01 (.01)	.03 (.02)
Subjective Social Status: Parent	.09** (.03)	.08** (.02)	.05** (.02)	.01 (.01)	.02 (.02)
Food Security: Parent	.06** (.02)	.06** (.02)	.05** (.02)	.01 (.02)	.03 (.02)
Parental Years of Education	.10** (.03)	.08** (.02)	.04** (.02)	.01 (.01)	.05* (.02)
Household Income	.11** (.03)	.08** (.02)	.05** (.02)	.01 (.01)	.02 (.01)
Administrative (Wave 1)^a^					
Family Economic Disadvantage	.11** (.03)	.08** (.02)	.05* (.02)	.01 (.01)	.02 (.02)
Neighborhood SES	.07** (.02)	.07** (.02)	.05* (.02)	.01 (.01)	.04* (.02)
Model with sex and minority status only	.06** (.02)	.06** (.02)	.04** (.02)	.01 (.01)	.02 (.01)

*Note*. All models include covariates for sex and minority status;

** *p* < .01,

* *p* < .05.

^a^ Although administrative data were collected at Wave 1, only adolescents with data available at Wave 3 were included in analyses.

#### Grades

We first examined whether adolescent-reported *subjective SES* predicted adolescents’ self-reported past-year academic performance. The model including sex, minority status, and adolescent-reported *subjective SES* at Wave 3 accounted for 9% of the variance in adolescent-reported grades at Wave 4. This *R*^*2*^ is comparable to those associated with models that included parent-reported subjective SES (*R*^*2*^ = .08), as well as parental years of education, household income, and family economic disadvantage (*R*^*2*^s: .10-.11).

The model including sex, minority status, and adolescent-reported *subjective social status* at Wave 3 accounted for 7% of the variance in adolescent-reported grades at Wave 4. Again, we found this *R*^*2*^ to be comparable to those associated with models that included parent-reported subjective social status (*R*^*2*^ = .09), as well as parental years of education, household income, and family economic disadvantage (*R*^*2*^s = .10-.11).

When we included adolescent-reported *food security* at Wave 3 in a model with sex and minority, the model accounted for 6% of the variance in adolescent-reported grades at Wave 4. This *R*^*2*^ is comparable to with the value for models including parent-reported food security (*R*^*2*^ = .06), but smaller than models with parental years of education, household income, and family economic disadvantage (*R*^*2*^s = .10-.11). See S5 Table for complete regression results for grades.

#### General health

The model including adolescent-reported *subjective SES* in addition to sex and minority status accounted for 8% of the variance in adolescent-reported general health at Wave 4, which is comparable to the *R*^2^ estimates for models including parent-reported subjective SES (*R*^*2*^ = .07), as well as parental years of education, household income, and family economic disadvantage (all *R*^*2*^s = .08).

Similarly, the model including sex, minority status, and adolescent-reported *subjective social status* accounted for 9% of the variance in adolescent-reported general health at Wave 4, which again is comparable to the *R*^*2*^ estimates for models including parent-reported subjective social status (*R*^*2*^ = .08), as well as models with parental years of education, household income, and family economic disadvantage. The model with sex, minority, and adolescent-reported *food security* accounted for 6% of the variance in adolescent-reported general health at Wave 4, which is comparable to the *R*^*2*^ estimates for models including parent-reported subjective food security (*R*^*2*^ = .06), as well as parental years of education, household income, and family economic disadvantage. See S5 Table for complete regression results for general health.

#### Psychological distress

The model including adolescent-reported *subjective SES*, sex, and minority status also predicted a similar proportion of variance in Wave 4 adolescent-reported psychological distress relative to parent-reported measures and administrative data, accounting for 6% of the variance compared to 4–5% for models including parent-reported subjective SES, parental years of education, household income, family economic disadvantage, and median neighborhood income. Models including sex, minority status, and adolescent-reported *subjective social status* and *food security* also predicted a similar proportion of variance in Wave 4 adolescent-reported psychological distress relative to parent-reported measures and administrative data, accounting for 6% of the variance compared to 5% for models including parent-reported subjective social status and parent-reported food security and 4–5% for all other variables. See S5 Table for complete regression results for psychological distress.

#### Problem behavior

Across adolescent-reported measures, parent-reported measures, and administrative data, no model including any measure of SES accounted for a statistically significant proportion of variance in Wave 4 adolescent-reported problem behaviors. Complete regression results for problem behavior appear in S5 Table.

#### Alcohol use

None of the models including adolescent-reported measures or parent-reported measures of subjective SES accounted for a statistically significant proportion of variance in Wave 4 adolescent-reported past-year alcohol use. However, a model with one parent-reported objective measure—household income—and a model including one administrative data predictor—neighborhood SES—accounted for 5% and 4% of the variance in adolescent reports of alcohol use, respectively. See S5 Table for complete regression results for alcohol use.

### Does prediction of outcomes vary by adolescent age?

**Fig 1:**
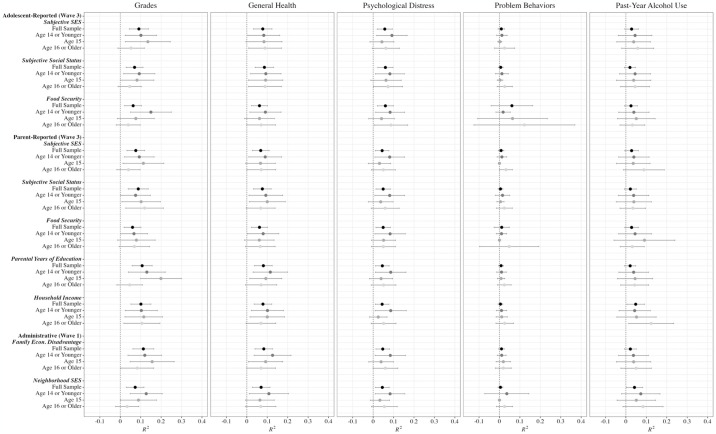
*R*^*2*^ values for predicting Wave 4 outcomes from SES measures by age group. Regression results for predicting Wave 4 outcomes from each SES measure for the full sample and by age group. Bars represent standard errors.

Finally, we examined whether the strength of prediction by adolescent-reported SES measures varied across age groups. Fig 1 displays *R*^*2*^ values and standard errors for the outcome variables—excluding problem behavior, as no SES measures were significant predictors of it—for each SES measure within the three age groups: age 14 and younger, age 15, and age 16 and older. Across the outcome variables, patterns of significance were similar to the full sample, as well as parent-reported and administrative indicators, with a few exceptions. For grades, *adolescent-reported subjective SES* was a significant predictor for adolescents 14 or younger and adolescents age 15 (*R*^*2*^s = .10 and .14, respectively), but not for adolescents 16 or older (*R*^*2*^ = .05). This pattern was replicated for *parent-reported subjective SES*, but not for the other subjective SES measures. Similarly, for general health, the model including *adolescent-reported food security* only explained a significant proportion of variance for adolescents 14 or younger (*R*^*2*^ = .09), as was true for *parent-reported food security* (*R*^*2*^ = .08). We observed similar patterns for psychological distress, wherein models including *adolescent-reported subjective SES*, *parent-reported subjective SES*, and *parent-reported food security* only predicted significant variance in the youngest age group (*R*^*2*^s ranged from .08-.09; *ns* for other age groups), and for alcohol use, where models including *parent-reported household income*, *family economic disadvantage*, and *neighborhood SES* were only significant predictors in the youngest age group (*R*^*2*^s ranged from .03-.05; *ns* for other age groups). See S6 Table for complete regression results for each outcome by age group.

## Discussion

We examined whether adolescent reports of subjective SES can serve as suitable proxies for parent reports of objective and subjective SES, as well as indicators of SES in administrative data. Using data from a sample of adolescents and their parents, we find moderate-to-strong associations between nearly all adolescent reports of subjective SES and parent reports of objective SES, replicating and extending prior work showing moderate-to-strong associations between parent and adolescent reports of SES [25], as well as a recent meta-analysis finding moderate correlations between subjective and objective SES measures [13]. Adolescent-reported subjective SES measures were further moderately correlated with parent-reported subjective SES and administrative data, suggesting that, in the absence of adolescent-reported objective SES, parent reports, or administrative data, adolescent reports of subjective SES—especially a single item tapping family subjective SES and the commonly-used ladder assessing subjective social status—are useful approximations of family socioeconomic status. Importantly, patterns of correlations were similar across age groups, demonstrating that younger and older adolescents alike could report on subjective SES in ways that approximate parent reports.

Patterns of correlations were mostly similar across economic disadvantage, as shown in S3 Table; correlations between adolescent-reported, parent-reported, and administrative data were not higher among adolescents from wealthier families. One notable exception is that correlations between adolescent-reported food security and many of the other SES measures failed to reach significance particularly for adolescents who were economically disadvantaged, potentially because this variable reflects the receipt of free and reduced lunch, which may skew perceptions of food (in)security. For example, previous work has shown greater discordance between child and parent perceptions of food security among recipients of SNAP benefits [38] Taken together with the smaller correlations we observed between adolescent-reported food security and most other SES measures in full sample and the smaller comparative *R*^2^ values for models including food security, this measure of adolescent SES was, in our sample, a less suitable proxy for parent and administrative data compared to adolescent subjective SES and social status.

Beyond simple correlations between adolescent reports of subjective SES, parent reports of objective and subjective SES, and administrative indicators of SES, we also examined whether adolescent-reported subjective SES predicted variance in adolescent well-being outcomes to the same extent as parent reports and administrative indicators. Self-reported adolescent subjective SES predicted variance in three well-being outcomes—past-year grades, general health, and psychological distress—as well or better than parent-reported objective and subjective SES and administrative data on SES. In these predictive models, we again find no consistent evidence that reports by older adolescents are better as proxies of parent reports than reports by their younger counterparts. Adolescent reports of subjective SES predicted little variance in problem behaviors and alcohol use, but the same was generally true for parent reports and administrative data. The limited explanatory power we observed may be attributable to observed low base rates of problem behaviors and alcohol use in our sample—which was not an at-risk sample—potentially contributing to low power to detect these effects. However, much of the literature shows only small direct effects between socioeconomic status and problem behaviors or substance use [39], including during adolescence [9, 25, 40]. Another view of the low explanatory power of SES in predicting adolescent problem behavior suggests that the effects of SES on substance use and problem behavior are complex, potentially operating in combination with other mechanisms such as neighborhood instability [41], family stress, and parental investment [42].

Taken together, our findings suggest that adolescents’ self-reports of subjective SES approximate parent reports of both subjective and objective SES, as well as administrative indicators of SES. Given previous work showing differential prediction of well-being outcomes from subjective and objective SES in adults and adolescents [9, 13], we add to the literature by leveraging a dataset with multiple indicators of subjective and objective SES across reporters, showing that adolescent self-reported subjective SES performs similarly to both parent-reported subjective SES indicators and objective reports of SES from parents and administrative indicators of SES. Notably, we find that the single-item measure of adolescent subjective SES showed strong correlations with other measures of SES, including parent and administrative data, and explained variance in well-being outcomes in a model together with sex and minority status. These findings illustrate that, in the absence of adolescent-reported objective SES indicators, parent-reported data, or administrative records, adolescents’ subjective reports of SES may be valid sources of information about family socioeconomic status. Collecting data from both parents and adolescents can be costly and time-intensive. Our findings suggest that researchers lacking resources to collect data from parents or access relevant administrative data can use adolescents’ subjective ratings of family SES with little loss when the focus is adolescent well-being. Though our goal was to examine how these adolescent-reported subjective measures perform vis-à-vis parent reports and administrative data with respect to our measured well-being outcomes, we agree with other work suggesting that objective and subjective measures of SES provide insight into different aspects of SES [43]. Further, our findings suggest that an age-appropriate single-item measure can be used to assess adolescent SES, which may be especially useful for researchers who are lacking space in study protocols or who may be using data collection methods (e.g., mobile phone or app-based methods; intensive repeated assessments) that favor brief, simple measures.

Although we reach these conclusions using a dataset with ten indicators of SES from a reasonably representative adolescent sample, our study is not without limitations. Though we tested for differences across age groups, our sample was restricted in its age range; 99% of the sample ranged from ages 13–17 at Wave 3. Future studies could extend our work by examining how well self-reports of SES from younger adolescents—ages 10–12—approximate reports from their parents. By restricting our analysis sample to adolescents with available data at Wave 3, it is possible that attrition from the study could have influenced our results. Although we used FIML to address missingness in our data, attrition could nevertheless have contributed to our findings. However, we are encouraged that was not the case, as, even considering attrition from the study, 51% of our final analysis sample experienced economic disadvantage, and participants without follow-up data at Wave 3 were less likely to be economically disadvantaged (38%) compared to those with Wave 3 data. And, although the analysis sample experienced slightly less economic disadvantage relative to the underlying population (55% economically disadvantaged), it was closer to the population than the original sample at Wave 1 (45% economically disadvantaged). Although our sample was reasonably representative with respect to SES, it was still comprised of many white (57%) adolescents in a single U.S. state. Additional work is needed to assess whether these findings would extend across locations and demographics. Finally, we saw little evidence for predictive associations between adolescent-reported subjective SES and problem behaviors and alcohol use, likely due to low rates of these self-reported behaviors in our sample. Here too, it is possible that attrition may have contributed to our findings, whereby adolescents who became involved in problem behaviors and alcohol use may have decided not to participate in later waves of the study. Further research is necessary to determine whether adolescent reports of subjective SES might predict problem behaviors later in life, mechanisms through which SES may have indirect effects, or in samples at high risk for substance use problems.

## Conclusion

Across ten measures of socioeconomic status, we find that adolescent-reported subjective SES correlates moderately with and predicts adolescent well-being outcomes to the same degree as (1) parent-reported subjective SES, (2) parent-reported objective SES, and (3) administrative data. These findings suggest that adolescent reports of subjective SES—including a single-item measure—serve as a suitable alternative to parent reports of objective and subjective SES, as well as SES indicators in administrative data. We found no consistent age differences, suggesting that even younger adolescents’ reports of SES should function similarly to their older counterparts as substitutes for parent reports or administrative data. The findings support the use of age-appropriate adolescent reports of subjective SES when parent reports and relevant administrative data are not available. Additional research is necessary to examine whether adolescent-reported subjective SES predicts well-being and problematic outcomes later in life and in higher-risk samples.

## Supporting information

S1 Table Demographics by Wave, compared to population. Demographics for population, Wave 1, and Wave 3.
(DOCX)

S2 Table Food security items. Unstandardized and standardized values for all adolescent- and parent-reported food security items.
(DOCX)

S3 Table Correlations among SES variables by economic disadvantage status. Pearson correlation coefficients among all SES variables by economic disadvantage status.
(DOCX)

S4 Table Pearson correlation coefficients between SES variables and well-being outcomes. *r*s and *n*s for all SES variables and all well-being outcomes.
(DOCX)

S5 Table Full regression results for all regression models. *R*^*2*^ values, standard errors, and p-values for all regression models.
(XLSX)

S6 Table Full regression results by age group. *R*^*2*^ values, standard errors, and p-values for all regression models by age group.
(XLSX)
