# Supplementary material for: Adolescent reports of subjective socioeconomic status: An adequate alternative to parent-reported objective and subjective socioeconomic status?
Source: PLoS One. 2025 Jan 17;20(1):e0317777. doi: 10.1371/journal.pone.0317777 (PMC11741571; doi:10.1371/journal.pone.0317777)
Supplement: S1 Table — Demographics for population, Wave 1, and Wave 3. (DOCX) [file pone.0317777.s001.docx]

**S1 Table.** *Demographics by Wave, Compared to Population*

| Demographic | Population  (*N* = 460,760) | Wave 1  (*N* = 2,104;  *M*_age_ = 12.36) | Wave 3  (*N* = 702;  *M*_age_ = 15.05) | No Wave 3 data available  (*N* = 1,402;  *M*_age_ = 15.28) | Test Statistic  Wave 3 Data vs. no Wave 3 Data^d^  [test statistic (df)] |
| --- | --- | --- | --- | --- | --- |
| Sex |  |  |  |  | 4.66^*^ (1) |
| Male | 51.38% | 47.91% | 44.59% | 49.57% |  |
| Female | 48.62% | 52.09% | 55.41% | 50.43% |  |
| Race/ethnicity^a^ |  |  |  |  | 27.13^**^ (6) |
| White | 51.34% | 52.14% | 57.04% | 49.67% |  |
| Black | 25.56% | 23.04% | 22.84% | 23.14% |  |
| Hispanic | 15.04% | 14.50% | 2.73% | 7.16% |  |
| Asian | 2.80% | 3.46% | 3.74% | 3.33% |  |
| American Indian | 1.32% | 2.07% | 2.44% | 1.88% |  |
| Pacific Islander | 0.10% | 0.19% | 0.29% | 0.14% |  |
| Multiracial | 3.83% | 13.42% | 10.92% | 14.68% |  |
| Economically disadvantaged | |  |  |  | 33.68^**^ (1) |
| No | 44.65% | 54.64% | 48.98% | 62.43% |  |
| Yes | 55.35% | 45.05% | 51.02% | 37.57% |  |
| Rural^b^ |  | 32.89% | 30.19% | 34.24% | 3.47 (1) |
| Mean neighborhood income (*SD*)^c^ | | $53,996.43 (29,342.55) | $56,869.58 (32,045.65) | $52,558.83 (27,792.63) | -3.18^**^ (2097) |

*Note*. ^a^ Multiracial was mutually exclusive for population only. Sample totals sum to > 100% for comparison with population. ^b^ Urban/rural Census designations unavailable matched at the population level. 34% of North Carolina’s population was rural based on 2010 Census data. <https://www.ncdemography.org/2021/09/20/why-rural-is-hard-to-define/> ^c^ Median neighborhood income estimates from Census data unavailable matched at the population. The median neighborhood income in the state of North Carolina from 2010-14 ACS estimates was $52,413. <https://www.census.gov/acs/www/data/data-tables-and-tools/narrative-profiles/2014/report.php?geotype=state&state=37> ^d^ Chi square tests were used for all demographic characteristics except mean neighborhood income which was tested using a t-test. ^**^ *p* < .01, ^*^ *p* < .05.
