# Supplementary material for: Adolescent reports of subjective socioeconomic status: An adequate alternative to parent-reported objective and subjective socioeconomic status?
Source: PLoS One. 2025 Jan 17;20(1):e0317777. doi: 10.1371/journal.pone.0317777 (PMC11741571; doi:10.1371/journal.pone.0317777)
Supplement: S2 Table — Unstandardized and standardized values for all adolescent- and parent-reported food security items. (DOCX) [file pone.0317777.s002.docx]

**S2 Table.** *Food Security Items*

|  |  | Original Items | | |  | Standardized Items | | |
| --- | --- | --- | --- | --- | --- | --- | --- | --- |
|  | *n* | Mean (*SD*) | Min | Max |  | Mean (*SD*) | Min | Max |
| **Adolescent-Reported Food Security Items** |  |  |  |  |  |  |  |  |
| In the past year, were you ever hungry, but didn't eat because you/your family could not afford enough food? (0 = *yes*, 1 = *no*) | 701 | 0.99 (0.12) | 0.00 | 1.00 |  | .00 (1.00) | -8.31 | 0.12 |
| In the past year, did you ever eat less than you felt you should because there wasn't enough money to buy food? (0 = *yes*, 1 = *no*) | 702 | 0.95 (0.22) | 0.00 | 1.00 |  | .00 (1.00) | -4.36 | 0.23 |
| How often in the past year did you/your family not have enough money to buy food? (1 = *often* to 4 = *never*) | 702 | 3.74 (0.60) | 1.00 | 4.00 |  | .00 (1.00) | -4.59 | 0.44 |
| How often in the past year could you not afford to eat balanced meals? (1 = *often* to 4 = *never*) | 702 | 3.71 (0.64) | 1.00 | 4.00 |  | .00 (1.00) | -4.21 | 0.46 |
| **Parent-Reported Food Security Items** |  |  |  |  |  |  |  |  |
| Over past year, the food that I bought just didn't last, and I didn't have money to get more. (0 = *sometimes or often true*, 1 = *never true*) | 609 | 0.98 (0.14) | 0.00 | 1.00 |  | .00 (1.00) | -5.78 | 0.17 |
| Over past year, I couldn't afford to eat balanced meals. (0 = *sometimes or often true*, 1 = *never true*) | 601 | 0.96 (0.20) | 0.00 | 1.00 |  | .00 (1.00) | -4.44 | 0.22 |
| In the last 12 months, did you ever cut the size of your meals or skip meals because there wasn't enough money for food? (0 = *yes*, 1=*no*) | 607 | 0.88 (0.33) | 0.00 | 1.00 |  | .00 (1.00) | -2.58 | 0.39 |
| In the last 12 months, did you ever eat less than you felt you should because there wasn't enough money for food? (0 = *yes*, 1 = *no*) | 607 | 0.87 (0.33) | 0.00 | 1.00 |  | .00 (1.00) | -2.53 | 0.40 |
| In the last 12 months, were you ever hungry but didn't eat because there wasn't enough money for food? (0 = *yes*, 1 = *no*) | 607 | 0.92 (0.26) | 0.00 | 1.00 |  | .00 (1.00) | -3.29 | 0.30 |
