# Supplementary material for: Adolescent reports of subjective socioeconomic status: An adequate alternative to parent-reported objective and subjective socioeconomic status?
Source: PLoS One. 2025 Jan 17;20(1):e0317777. doi: 10.1371/journal.pone.0317777 (PMC11741571; doi:10.1371/journal.pone.0317777)
Supplement: S3 Table — Pearson correlation coefficients among all SES variables by economic disadvantage status. (DOCX) [file pone.0317777.s003.docx]

**S3 Table.** *Correlations Among SES Variables by Economic Disadvantage Status (values above diagonal = economically disadvantaged; values below diagonal = not economically disadvantaged)*

|  | Adolescent-Reported | | |  | Parent-Reported | | | | |  | Admin. |
| --- | --- | --- | --- | --- | --- | --- | --- | --- | --- | --- | --- |
|  | 1 | 2 | 3 |  | 4 | 5 | 6 | 7 | 8 |  | 9 |
| 1. Subjective SES: Adolescent |  | .51^**^  (349) | .20^**^  (350) |  | .34^**^  (315) | .33^**^  (315) | .17^**^  (314) | .14^**^  (340) | .34^**^  (326) |  | .16^**^  (349) |
| 2. Subjective Social Status: Adolescent | .41^**^  (329) |  | .14^**^  (349) |  | .33^**^ (315) | .42^**^  (315) | .13^*^  (314) | .19^**^  (339) | .41^**^ (326) |  | .28^**^  (348) |
| 3. Food Security: Adolescent | .28^**^  (329) | .22^**^ (336) |  |  | .05 (315) | .14^*^  (315) | .10  (314) | .02  (340) | .08 (326) |  | -.02  (349) |
| 4. Subjective SES: Parent | .38^**^  (275) | .20^**^ (280) | .15^*^  (280) |  |  | .52^**^  (314) | .24^**^  (314) | .16^**^  (315) | .42^**^ (303) |  | .28^**^  (314) |
| 5. Subjective Social Status: Parent | .27^**^  (275) | .36^**^ (280) | .14^*^  (280) |  | .39^**^ (278) |  | .21^**^  (313) | .28^**^  (315) | .46^**^ (303) |  | .31^**^  (314) |
| 6. Food Security: Parent | .25^**^  (274) | .15^*^ (279) | .30^**^  (279) |  | .36^**^ (278) | .25^**^  (276) |  | .12^*^  (314) | .20^**^ (302) |  | .11^*^  (313) |
| 7. Parental Years of Education | .15^**^  (304) | .14^*^ (311) | .11  (311) |  | .14^*^ (278) | .24^**^  (278) | .10  (277) |  | .34^**^ (326) |  | .18^**^  (339) |
| 8. Household Income | .31^**^  (296) | .22^**^ (303) | .16^**^  (303) |  | .40^**^ (274) | .30^**^  (273) | .31^**^  (273) | .28^**^  (302) |  |  | .34^**^  (325) |
| 9. Neighborhood SES | .10  (328) | .10  (335) | .10  (335) |  | .10  (279) | .03  (279) | .14^*^  (278) | .14^*^  (310) | .38^**^ (302) |  |  |

*Note.* ^**^ *p* < .01, ^*^ *p* < .05.
