# Supplementary material for: Adolescent reports of subjective socioeconomic status: An adequate alternative to parent-reported objective and subjective socioeconomic status?
Source: PLoS One. 2025 Jan 17;20(1):e0317777. doi: 10.1371/journal.pone.0317777 (PMC11741571; doi:10.1371/journal.pone.0317777)
Supplement: S4 Table — rs and ns for all SES variables and all well-being outcomes. (DOCX) [file pone.0317777.s004.docx]

**S4 Table.** *Pearson Correlation Coefficients (n) between SES Variables and Well-Being Outcomes*

|  | Grades | General Health | Psychological Distress | Problem Behavior | Past-Year Alcohol Use |
| --- | --- | --- | --- | --- | --- |
| Adolescent SES Measures |  |  |  |  |  |
| Subjective SES: Adolescent | .20^**^  (527) | .13^**^  (548) | -.10^*^  (549) | -.07  (547) | .08  (549) |
| Subjective Social Status: Adolescent | .12^**^  (231) | .17^**^  (552) | -.13^**^  (553) | -.04  (551) | .02  (553) |
| Food Security: Adolescent | .10^*^  (532) | .05  (553) | -.11^*^  (554) | -.22^**^  (552) | -.03  (554) |
| Parent SES Measures |  |  |  |  |  |
| Subjective SES: Parent | .15^**^  (479) | .10^*^  (496) | -.02  (496) | -.06  (494) | .08  (496) |
| Subjective Social Status: Parent | .17^**^  (480) | .13^**^  (496) | -.08  (496) | .01  (494) | .03  (496) |
| Food Security: Parent | .06  (477) | .06  (494) | -.07  (494) | -.08  (492) | .07  (494) |
| Parental Years of Education | .26^**^  (520) | .16^**^  (540) | -.02  (540) | -.06  (539) | .04  (541) |
| Household Income | .25^**^  (504) | .18^**^  (525) | -.01  (525) | -.02  (523) | .17^**^  (525) |
| Administrative Data |  |  |  |  |  |
| Family Economic Disadvantage | .29^**^  (521) | .19^**^  (542) | -.04  (543) | -.08^*^  (541) | .08  (543) |
| Neighborhood SES | .17^**^  (531) | .13^**^  (552) | -.03  (553) | .01  (551) | .15^**^  (553) |

*Note.* ^**^ *p* < .01, ^*^ *p* < .05.
